# Dietary Inflammatory Index and Risk of Breast Cancer Based on Hormone Receptor Status: A Case-Control Study in Korea

**DOI:** 10.3390/nu11081949

**Published:** 2019-08-19

**Authors:** Seohyun Lee, Arlene Lansangan Quiambao, Jeonghee Lee, Jungsil Ro, Eun-Sook Lee, So-Youn Jung, Mi-Kyung Sung, Jeongseon Kim

**Affiliations:** 1 Graduate School of Cancer Science and Policy, National Cancer Center, Goyang-si, Gyeonggi-do 10408, Korea; 2 Center for Breast Cancer, Research Institute and Hospital, National Cancer Center, Goyang-si, Gyeonggi-do 10408, Korea; 3 Department of Food and Nutrition, Sookmyung Women’s University, Seoul 04310, Korea

**Keywords:** Dietary Inflammatory Index, breast cancer, hormone receptor, Korea

## Abstract

Breast cancer is the most common cancer in women globally, and the risk of developing breast cancer is associated with inflammation. The present study aimed to examine the association between the Dietary Inflammatory Index (DII^®^) and breast cancer in Korean women and investigate whether the tumor’s hormone receptor status affects this association. In this case-control study, we enrolled 364 breast cancer patients and 364 age-matched controls. DII scores were calculated from dietary intake evaluated by a 106-item food frequency questionnaire. The DII score was significantly higher in cases than in controls. After adjusting for potential confounders, the odds ratio (OR) of breast cancer was higher in the highest DII tertile (OR = 3.68, 95% confidence interval (CI): 2.34–5.80, *p* for trend < 0.0001) than in the lowest tertile. We found that higher DII scores were related to an increased risk of breast cancer for estrogen receptor (ER)+/progesterone receptor (PR)+ tumors regardless of menopausal status (OR = 2.59, 95% CI: 1.37–4.88 in the highest DII category, *p* for trend = 0.01 for premenopausal women; OR = 11.00, 95% CI: 2.93–41.30 in the highest DII category, *p* for trend = 0.0004 for postmenopausal women), but not for ER−/PR− status. Our results suggested that the DII scores are positively associated with breast cancer risk in Korean women and that this relationship is more robust in ER+/PR+ tumors.

## 1. Introduction

Breast cancer is the most common cancer and is one of the main causes of cancer mortality in women globally. Based on the 2018 GLOBOCAN estimates, approximately 2.1 million new breast cancer cases and 0.6 million breast cancer deaths occurred worldwide [1]. The number of new breast cancer patients increased approximately 1.5-times in 2018 [1], compared to the 1.4 million newly diagnosed cases in 2008 [2]. A recent study estimated that in Korea, breast cancer is the most frequently diagnosed cancer in 35–64-year-old women, and the most common cancer of all newly diagnosed cases among women in 2019 [3]. Thus, breast cancer is a major emerging health problem in Korean women.

Estrogen receptors (ERs) and progesterone receptors (PRs) are hormone receptors found on breast cells that pick up hormone signals resulting in cell growth [4,5]. Breast cancer can be classified as ER and/or PR expression, and the clinical, pathological, and molecular characteristics of breast cancer differ depending on the type of hormone receptor tumor [6]. Thus, knowing the hormone receptor status can be an important factor in determining effective treatment options. Epidemiologic data have shown that ER-positive (ER+) or PR-positive (PR+) tumors exhibit a stronger clinical response to hormone therapy than ER-negative (ER−) or PR-negative (PR−) breast cancer [7]. Previous studies have also shown that there may be differences in the association of breast tumor subtypes with epidemiologic risk factors. The effects of several risk factors for breast cancer, such as parity, body mass index (BMI), and dietary status, seem to differ based on ER and PR status [8,9]. Among these risk factors, dietary factors, including legumes, fruit, vegetables, and nutrients, have inconsistent protective effects against breast cancer [10,11]. The inconsistent results regarding various risk factors for breast cancer may be due to a lack of controlling for these hormone receptors. Hence, it is important to identify the hormone receptor status of breast tumors with various risk factors for breast cancer.

Chronic inflammation is an important contributor to the development and progression of cancers, including breast cancer. Chronic inflammation can cause DNA damage and stimulate chronic cell proliferation, which are important factors in carcinogenesis [12,13]. A large body of evidence indicates that circulating inflammatory biomarkers, including interleukin (IL)-4, IL-6, IL-8, C-reactive protein (CRP), and tumor necrosis factor-α (TNF-α), may be associated with breast cancer risk [14,15,16]. Epidemiologic evidence indicates that diet influences chronic inflammation [17,18]. Dietary patterns high in fruits, vegetables, whole grains, and fish have been inversely associated with levels of inflammatory markers [19]. In contrast, dietary patterns with high levels of refined starches, sugar, red meat, saturated fat, and trans-fatty acids may be associated with activation of the innate immune system [18].

The Dietary Inflammatory Index (DII^®^), a literature-derived and population-based dietary scoring system, was recently developed to assess the inflammatory potential of the diet [20]. Previous studies have shown an association between DII scores and inflammatory cytokines, including CRP and IL-6 [21,22,23]. To date, a few epidemiologic studies have examined the association between DII scores and the incidence of breast cancer [24,25,26] and breast cancer mortality [27]. Nevertheless, the association between DII and risk of breast cancer in Korean women remains largely unknown. Therefore, the main objective of this study was to investigate whether a higher DII score, reflecting a more proinflammatory diet, is associated with increased breast cancer risk in Korean women. Furthermore, we assessed the relationship between DII and the risk of breast cancer among premenopausal women and postmenopausal women with the disease defined by hormone receptor status.

## 2. Materials and Methods

### 2.1. Data Source and Study Population

Initially, a total of 441 breast cancer patients were recruited among patients who were admitted for surgery at the Center for Breast Cancer, National Cancer Center Hospital (NCC) in the Republic of Korea, from July 2007 to September 2008. Among those patients, 415 patients agreed to participate in the study. We excluded participants with previous histories of other cancers, those who were unable to cooperate in the interview, and women who reported implausible daily energy intake (<500 or >4000 kcal/day). After those participants were excluded, 398 patients were eligible for the analysis.

**Figure 1:**
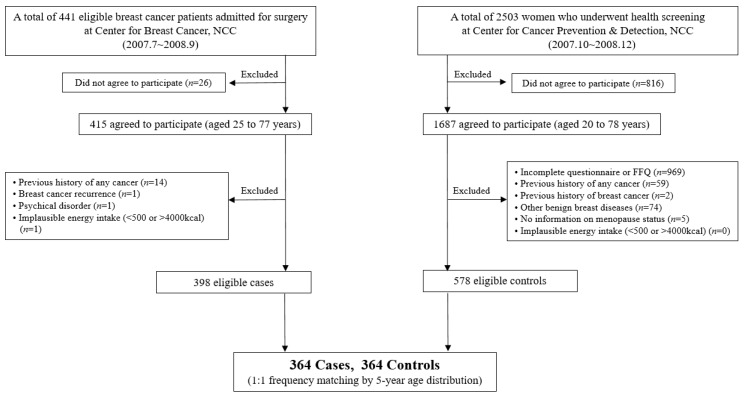
Flow chart of the study population selection.

The 2503 cancer-free controls were recruited among women who received regular health check-ups at the Center for Early Detection and Prevention at the same hospital between October 2007 and December 2008. Among those women, 1687 agreed to participate in the study. Based on eligibility criteria, we excluded 1109 subjects with insufficient dietary intake information, a history of malignant neoplasms or other benign breast diseases, a lack of information on menopausal status, or implausible self-reported daily energy intake (<500 or >4000 kcal/day). After exclusion, 578 women were eligible for inclusion in the control group, and the controls were frequency-matched to cases within a 5-year age distribution. Ultimately, data from 728 subjects (364 cases and 364 controls) were included in the final data analysis (Figure 1). Informed content was obtained from all participants, and the study protocol and consent forms were approved by the NCC Institutional Review Board (IRB number: NCCNCS 07-083).

### 2.2. Dietary Assessment and Calculation of the DII

The dietary intake of the participants was assessed using a 106-item semi-quantitative food frequency questionnaire (SQFFQ). The reliability and validity of the SQFFQ have been previously reported [28]. Subjects provided their individual average frequency intake of specific foods and the typical portion sizes of their meals in the year preceding the interview.

To calculate DII scores, dietary assessment data from the SQFFQ were used. The method was originally devised by Cavicchia et al. [29], and was updated by Shivappa et al. [20]. Dietary data were compared with the representative world database that provided the estimated mean and standard deviation (SD) for daily intake of each parameter of the DII from 11 countries. A standard global mean for each parameter from the world database was subtracted from the participants’ intake and divided by its SD to generate Z-scores. These Z-scores were converted to percentiles, minimizing the effects of the problem of right skewing of the data. The percentiles were then multiplied by 2, and 1 was subtracted to achieve a symmetrical distribution with values concentrated on 0. After these steps, the resulting value was multiplied by the corresponding inflammatory effect score for each parameter, and the parameter-specific DII scores were summed to obtain the overall DII score.

A higher DII score indicated a higher intake of proinflammatory foods, while a lower score showed a higher consumption of anti-inflammatory foods [25]. In the current study, we included the following 37 food items to compute the DII score: carbohydrates, protein, total fat, monounsaturated fatty acids (MUFAs), polyunsaturated fatty acids (PUFAs), saturated fat, ω-3 fatty acid, ω-6 fatty acid, cholesterol, fiber, vitamin B_1_, vitamin B_2_, niacin, vitamin B_6_, vitamin B_12_, folic acid, vitamin A, vitamin C, vitamin D, vitamin E, β-carotene, iron, magnesium, selenium, zinc, flavan-3-ol, flavones, flavonols, flavonones, anthocyanidins, isoflavones, garlic, ginger, onion, green tea, pepper, and alcohol. Energy was not used to compute the DII because all components of the DII were adjusted for energy intake using the energy density approach, which was calculated per 1000 kcal of energy [30]. The world database was also standardized to 1000 kcal/day [31,32,33].

### 2.3. Other Measurements

Sociodemographic, lifestyle, reproductive, and medical history data were collected using a structured questionnaire by a trained dietitian at the time of initial recruitment prior to cancer diagnosis. Education level was categorized into four groups: ≤elementary school, middle school, high school, or >high school. Occupation was divided into four groups: housewife, profession or office worker, sales or service, or others. Smoking status was classified as past/never or current, and alcohol consumption was classified as never or ever. Physical activity was calculated using the short form (version 20. April 2004) of the International Physical Activity Questionnaire (IPAQ) and was summarized in units of metabolic equivalent (MET). Then, physical activity was classified into four categories (unit: MET-min/week): <396, 396–1272, 1272–2772, or ≥2772. Marital status was categorized as married, single, or others. Reproductive factors included age at menarche, parity, menopausal status, type of menopause, and postmenopausal hormone use. Information on the first-degree family history of breast cancer was also collected.

Anthropometric characteristics were also determined using standardized methods. Height and weight were measured to the nearest 0.1 cm and 0.1 kg, respectively. BMI, which reflects obesity status, was defined as weight (kg)/height (m^2^).

Breast cancer patients were diagnosed by biopsy, and breast cancer controls were performed through mammography [34]. ER and PR contents were analyzed in samples cut from formalin-fixed tissue sections. Paraffin-embedded breast tumors were analyzed by immunohistochemistry (Ventana Medical System, Tucson, AZ, USA). More detailed information concerning the immunohistochemistry procedure protocols is given elsewhere [35].

### 2.4. Statistical Analysis

Sociodemographic characteristics and lifestyle-related variables are expressed as the means with their SDs (continuous variables) or numbers with percentages (categorical variables). Differences between cases and controls were examined by Student’s *t*-test for continuous variables or the chi-square test for categorical variables. A linear residual regression method was used to adjust the dietary components in the DII for total energy intake [30]. The DII score was categorized into tertiles based on the distribution among the controls. To determine breast cancer risk according to DII score, unconditional logistic regression analysis was used to estimate the odds ratio (OR) and the 95% confidence intervals (95% CIs). Statistical models were based on controlling for covariates, which were significant between cases and controls, (i) Total population group: height, BMI, education level, occupational status, age at menarche, parity, and total energy intake, (ii) premenopausal women group: BMI, education level, physical activity (categorical), and parity, (iii) postmenopausal women group: BMI, education level, supplement use, age at menarche, parity, type of menopause, menopause hormone use, and total energy intake. A linear trend test across increasing categories of the DII was conducted with continuous variables using the median value in each category. All data were analyzed using SAS 9.4^®^ (SAS Institute, Cary, NC, USA). Two-sided *p*-values were considered to determine statistical significance.

## 3. Results

### 3.1. General Characteristics of the Study Population

The general characteristics of the study subjects are shown in Table 1. The mean ages of the case and control participants were not significantly different. Height, weight, and BMI were higher in the case group than in the control group. Overall, there were statistically significant differences between cases and controls in terms of education (*p* < 0.0001), occupation (*p* = 0.04), parity (*p* < 0.0001), and daily total energy intake (*p* = 0.01). In addition, age at menarche (*p* = 0.001), postmenopausal hormone use (*p* = 0.0004), and type of menopause (*p* = 0.02) were also significantly different between cases and controls in the group of postmenopausal women. The mean DII scores were 0.05 ± 2.59 and 1.03 ± 2.41 in the controls and cases, respectively (*p* < 0.0001), indicating a more proinflammatory diet in the case subjects.

### 3.2. Food and Nutrient Intake as Components of the DII of the Participants

Table 2 presents the intake of the 37 dietary components of the DII in cases and controls. In the control subjects, the intake of most dietary components of the DII, except for carbohydrates, flavones, flavonones, ginger, green tea, and alcohol, was significantly higher than in the cases. In particular, carbohydrate intake was significantly higher in the case group than in the control group (187.97 ± 20.01 g/day in control subjects vs. 195.74 ± 19.85 g/day in case subjects, *p* < 0.0001). No significant differences were observed between controls and cases in flavones, flavonones, ginger, and alcohol intake.

### 3.3. Association Between the DII Score and the Risk of Breast Cancer

In this study, a higher DII score was associated with an increased OR of breast cancer. Table 3 presents the OR and 95% CI for the risk of breast cancer according to tertiles of DII score. The crude OR for breast cancer in all participants was significantly higher in the highest DII group (OR = 2.98, 95% CI: 2.01*–*4.42 in the highest DII category, *p* for trend <0.0001) than in the lower tertile groups. After adjusting for potential confounders, we also observed a significant positive association between DII and the risk of breast cancer. Individuals in the highest tertiles showed a higher risk of breast cancer (OR = 3.68, 95% CI: 2.34–5.80 in the highest DII category, *p* for trend <0.0001) than those in the lowest tertiles. In the multivariate-adjusted model, the OR for breast cancer in the highest DII group of premenopausal women and postmenopausal women was 2.11 (95% CI: 1.21–3.67, *p* for trend = 0.01) and 6.13 (95% CI: 2.66–14.12, *p* for trend <0.0001), respectively, compared to the lowest DII group.

### 3.4. Association Between the DII Score and the Risk of Breast Cancer According to Hormone Receptor Status

The OR and 95% CI of breast cancer risk characterized by ER and PR status according to DII score are shown in Table 4. Among all subjects, participants with ER+ and PR+ (ER+/PR+) status had an increased risk of breast cancer in the highest tertile (OR = 4.29, 95% CI: 2.45–7.54 in the highest DII category, *p* for trend <0.0001) compared to that of individuals in the lowest tertile. After adjusting for confounding variables, the positive association was more pronounced in postmenopausal women with ER+/PR+ status (OR = 11.00, 95% CI: 2.93–41.30 in the highest DII category, *p* for trend = 0.0004). In addition, the multivariate-adjusted OR and 95% CI for breast cancer were higher in the highest DII group (OR = 5.00, 95% CI: 2.32–10.77 in the highest DII category, *p* for trend <0.0001) compared to those of the lowest tertile group with the ER− and PR− (ER−/PR−) status. However, no significant association was found between DII score and ER−/PR− status in premenopausal women.

## 4. Discussion

The present case-control study demonstrated the association between the inflammatory potential of diet, as measured by DII scores, and the risk of breast cancer among Korean women. We showed that DII scores were positively associated with breast cancer risk. These results support the hypothesis that women consuming a more proinflammatory diet may be at increased risk for breast cancer. The association between higher DII and an increased risk of breast cancer was also observed in patients with ER+/PR+ and ER−/PR− status (but not in premenopausal women).

Chronic inflammation is associated with cancer development, particularly in the context of breast cancer [12,13,36]. Oxidative DNA damage caused by chronic inflammation is one of the critical causes of mutations in carcinogenic genes [37]. A large body of evidence indicates that diet affects inflammation status, which plays a major role in the development of breast cancer [17,18]. The Mediterranean dietary pattern, characterized by being high in fruits, vegetables, whole grains, olive oil, and fish and low in red meat and butter, with moderate alcohol consumption, has been related to a lower level of inflammation [19]. In contrast, dietary patterns with high levels of refined starches, sugar, red meat, saturated fat, and trans-fatty acids, and low levels of fiber and natural antioxidants from whole grains, fruit, and vegetables may be associated with the activation of the innate immune system [18]. Dietary factors have been implicated in causing cancer, including breast cancer [38]. Therefore, the effects of dietary factors on breast cancer risk have been extensively studied globally. Previous studies on cancer epidemiology focusing on dietary factors in Koreans have shown that consumption of vegetables, mushrooms and soybean foods is negatively associated with breast cancer risk [11,39]. In addition, a case-control study reported that a vegetable- and seafood-rich dietary pattern might be inversely related to breast cancer risk in Korean women [40]. However, there is little evidence concerning the inflammatory effect of the overall diet on the risk of breast cancer among Koreans. Thus, an assessment of the association between the inflammatory potential of diet and breast cancer risk in Korean is meaningful.

The DII was recently developed as a tool to measure the inflammatory potential of the diet [20]. A high DII score is associated with high proinflammatory dietary component intake and low consumption of anti-inflammatory foods. We found that the mean DII score was higher in cases than in controls. In addition, the risk of breast cancer was positively associated with higher DII scores in this study. Thus far, the existing literature has shown inconsistent results regarding the relationship between the DII and breast cancer risk. Consistent with the results of this study, a positive association between increasing DII score and breast cancer risk has been reported in Italy [25], Iran [41], and China [24]. Additionally, a prospective study conducted in Sweden reported that a higher DII score is related to a higher risk of breast cancer. Conversely, no association between DII and breast cancer risk was observed in a case-control study from Germany [42] and a cohort study from the USA [27]. The inconsistency across studies concerning the DII and breast cancer risk might be attributed to the difference in the biology of breast cancer, study design, and the other underlying characteristics of each study population, such as race and lifestyle. Moreover, the conflicting results among studies may be due, in part, to differences in the availability food parameters for DII calculations because food consumption differs from country to country due to cultural differences and food availability. The current study used 37 dietary parameters that included anti-inflammatory foods, including garlic, onion, and pepper, as well as nutrient components to compute the DII score. A Chinese breast cancer case-control study [24] used 33 food parameters for DII calculation, and breast cancer studies in Italy [25] and Iran [41] used 31 parameters, including anti-inflammatory foods and flavonoids. On the other hand, other breast cancer studies used 28–32 DII components that did not include several anti-inflammatory parameters, such as garlic, pepper, and flavonoids [27,43]. The higher DII score in cases in our study means that breast cancer patients in the case group may have a more proinflammatory diet than controls, and the results may indicate that the more proinflammatory diet is linked with an increased risk of breast cancer in Korean females. Thus, our results provide evidence that diet-related inflammation is involved in the etiology of breast cancer.

Previous studies have evaluated the relationship between nutrients and certain food items and circulating inflammatory markers [44,45,46]. The nutrient and food parameters included in the DII were selected based on the effect of each parameter on anti-inflammation or proinflammation [20]. Thus, individual components of the DII are also linked with changes in inflammation markers. Anti-inflammatory foods such as garlic have been consumed in Korea for a long time. Garlic consumption has been related to cancer prevention effects by multiple mechanisms, such as the inhibition of DNA adduct formation, improvement in antioxidant defense and DNA repair, and decreased inflammation [47]. The present study showed that the intake of garlic was lower in patients with breast cancer than in healthy controls. On the other hand, we found that the intake of carbohydrates, which constitute an individual component of the DII, was higher in patients with breast cancer than in healthy controls. High consumption of carbohydrates is associated with a high insulin response that increases circulating proinflammatory cytokine levels [48]. A Korean study reported that higher carbohydrate intake is associated with blood total antioxidant capacity (TAC) as an oxidative stress-related indicator and a breast cancer risk factor [49]. Therefore, the high consumption of anti-inflammatory foods and the reduced proinflammatory food intake may have amplified anti-inflammatory potential that prevents the development of breast cancer.

Several mechanisms have been proposed to explain the effect of diet on breast cancer: the antioxidant effect [50], inhibition of cell proliferation and invasion [51], inhibition of autophagy [52], and reduction in endogenous estrogen production [53]. One of the most studied mechanisms is inflammatory change due to dietary factors. The potential role of chronic inflammation in breast cancer risk is supported by evidence from studies on the association between inflammatory markers and breast cancer incidence. Higher concentrations of many inflammatory markers, including CRP, IL-1β, IL-6, and TNF-a, have been linked with a higher risk of breast cancer [15,16,54] through the stimulation of angiogenesis, proliferation, migration, metastasis, and prevention of apoptosis. A recent study showed that serum IL-6, IL-8, and TNF-α were positively correlated with clinical tumor stage and lymph node status in Chinese breast cancer patients [55]. The DII was designed to take into account the relationship between each dietary parameter and inflammatory cytokines [20], and the existing literature reports that the DII is associated with changes in plasma inflammatory markers [21,56,57]. Shivappa et al. [21] have shown a positive association between the DII and CRP in American individuals, and Na et al. [58] also reported that a high DII was related to a high level of CRP in Korean individuals.

The literature published to date has suggested that the effects of diet on breast cancer may vary depending on several factors, such as menopausal status and molecular profiles of the tumor [6]. Although the differential relationship between breast cancer risk and menopausal status has been reported in previous studies [59], we could not find any significant difference by menopausal status in the association between breast cancer risk and the DII score. Nevertheless, the present study showed that the association of the DII with breast cancer was more noticeable in postmenopausal women than in premenopausal women. Postmenopausal estrogen deficiency has been shown to alter fat metabolism and promote breast cancer [60]. Obesity, due to alterations in fat metabolism, is one of the endocrine-associated risk factors for breast cancer in postmenopausal women, and it may be associated with increased estrogen production by aromatase activity in breast adipose tissue [61]. In this study, BMI was higher in postmenopausal women than in premenopausal women. Additionally, the proportion of obese (≥25 kg/m^2^ BMI) breast cancer patients was higher in the postmenopausal group than in the premenopausal group. In the current study, dietary inflammatory potential appeared to have a stronger impact on postmenopausal women who are prone to developing breast cancer.

The ER and PR status of breast cancers may explain the emergence of breast cancer subtypes with different etiologies [6,9]. A number of epidemiological studies have investigated the association of dietary factors with breast cancer subtypes due to hormone receptor status, but the results have not yet been conclusive. Previous studies have shown inconsistent associations between fruit, vegetable, and micronutrient intake and breast cancer risk based on ER status [10,62,63]. Breast cancer is more frequently ER+, approximately 75% of all breast cancers are of the ER+ status [64,65]. In addition, approximately 65% of patients with ER+ tumors are also PR+ [66]. Since the overall increase in breast cancer risk seems to be mainly due to an increased incidence of hormone receptor-positive tumors [67], targeted control of risk factors for hormone receptor-positive status in breast cancer may be one of the effective strategies to reduce overall breast cancer incidence. Our results showed a positive association between DII score and risk of breast cancer in Korean women with either ER+/PR+ or ER−/PR− statuses. Consistent with our results, a recent study of Chinese women reported that a higher DII score was linked with an increased risk of breast cancer with ER+/PR+ and ER−/PR− statuses [24]. However, this study showed that the abovementioned relationship varies depending on menopausal status. In postmenopausal women, the OR for breast cancer was related to the DII for both ER+/PR+ and ER−/PR− subtypes. Furthermore, the DII seemed to be more robustly correlated with breast cancer with ER+/PR+ tumors than with ER−/PR− tumors, whereas premenopausal women did not show this association; the breast cancer risk according to DII score was significantly linked only to ER+/PR+ tumors in premenopausal women. Thus, the DII may be a potential clue to reduce the risk of ER+/PR+ tumors regardless of menopausal status in Korean women, and the DII might be more intensively associated with breast cancer risk in postmenopausal women.

There are several strengths in the present study. To reduce errors in the collected data, data were gathered in face-to-face interviews to enable comprehensive information collection on related lifestyle factors, thus reducing the possibility of misclassification and measurement errors. In particular, to our knowledge, this study may be the first hospital-based case-control study to investigate whether the DII has a positive association with the risk of breast cancer according to the hormone receptor status of the tumor in Korean women. A number of studies have been conducted on the relationship between diet and breast cancer risk in the context of specific nutrients or foods [68]. However, studies on nutrients or foods may not be sufficient to assess the relationship between breast cancer and diet. Since people eat foods that are mixed with various nutrients and non-nutrients, these ingredients are absorbed by the body and then act in complex manners. Thus, it is necessary to assess the relationship between the overall diet and the prevention and treatment of diseases. Moreover, the DII is a reliable nutritional assessment tool that reflects the standardization of individual intakes to worldwide referent values based on an extensive review of the literature. Therefore, our findings provide useful and credible information for developing more appropriate strategies to prevent breast cancer in Korean women.

Despite these strengths, this study has several limitations. First, recall bias and selected bias may have affected the results because this is a case-control study. Generally, the cases might have better recall than the controls. In addition, the controls might have been more health-conscious than the cases, and hospital-based controls may have exaggerated their healthier habits compared to their community-based counterparts. Second, cancer patients may differ from controls in their recall of dietary habits. Therefore, the interviewer tried to collect information as soon as possible after diagnosis, which was typically right after surgery. Third, we used SQFFQ to estimate subjects’ dietary intake, which may result in an incorrect quantification of the actual consumption. Although well-trained interviewers performed the survey using a validated SQFFQ and pictures of portion sizes, measurement errors in dietary intake are inevitable. In addition, women may underestimate their dietary intake on self-reports because of social desirability [69]. For this reason, the dietary intake results should be interpreted with caution. Fourth, only 37 out of 45 original dietary inflammatory parameters were available for the DII calculation. Fifth, although many confounders have been adjusted in the present study, a wide range of unmeasured potential confounding factors still needs to be considered. Finally, the association between DII score and circulating inflammatory markers, such as CRP and IL-6, were not examined in the present study. This is because it is not appropriate to measure the habitual diet prior to disease onset and then measure inflammatory markers after diagnosis. In addition, it is difficult to establish the temporal sequence between the DII and levels of inflammatory biomarkers because of the case-control study design. Therefore, further longitudinal studies are needed to better understand the impact of the DII on blood inflammatory markers and breast cancer outcomes in Korean women.

## 5. Conclusions

In conclusion, the present study found that a more proinflammatory diet is positively associated with an increased risk of breast cancer in Korean women, especially those with some risk factors, such as postmenopausal status and hormone receptor status. In the future, large-scale prospective epidemiological studies are required to investigate whether the identified inflammation-related dietary factors affect circulating inflammatory markers, such as CRP, and circulating cytokine levels, and the development of breast cancer in Korean women.

## Figures and Tables

**Table 1:** General characteristics of the study population.

	Total (*n*= 728)			Premenopausal Women (*n* = 444)			Postmenopausal Women (*n* = 284)		
	Controls (*n* = 364)	Cases (*n* = 364)	*p-*Value	Controls (*n* = 223)	Cases (*n* = 221)	*p-*Value	Controls (*n* = 141)	Cases (*n* = 143)	*p-*Value
Age (year)	47.7 ± 7.9	47.8 ± 8.1	0.71	43.0 ± 4.4	43.2 ± 5.0	0.63	55.2 ± 6.3	54.1 ± 7.6	0.20
Height (cm)	158.0 ± 5.4	156.9 ± 5.4	0.01	158.6 ± 5.3	157.7 ± 5.2	0.08	156.9 ± 5.3	155.7 ± 5.5	0.05
Weight (kg)	56.8 ± 6.7	57.8 ± 8.2	0.10	56.3 ± 7.1	57.2 ± 8.6	0.23	57.7 ± 5.9	58.6 ± 7.5	0.23
BMI (kg/m^2^)	22.8 ± 2.5	23.5 ± 3.1	0.002	22.4 ± 7.1	23.0 ± 3.2	0.04	23.4 ± 2.1	24.2 ± 2.9	0.01
<25	306 (84.1)	265 (72.8)	0.0002	196 (87.9)	174 (78.7)	0.01	110 (78.0)	91 (63.6)	0.01
≥25	58 (15.9)	99 (27.2)		27 (12.1)	47 (21.3)		31 (22.0)	52 (36.4)	
First-degree family history of breast cancer (yes)	10 (2.8)	18 (5.0)	0.13	6 (2.7)	9 (4.1)	0.14	4 (2.9)	9 (6.3)	0.17
Supplement use (yes)	215 (59.9)	194 (53.4)	0.08	120 (54.3)	123 (55.9)	0.73	114 (82.6)	97 (67.8)	0.004
Education level			<0.0001			<0.0001			<0.0001
≤Elementary	21 (5.8)	65 (14.9)		5 (2.3)	8 (3.6)		16 (11.4)	46 (32.2)	
Middle school	20 (5.5)	45 (12.4)		6 (2.7)	25 (11.4)		14 (10.0)	20 (17.0)	
High school	131 (36.3)	169 (46.6)		71 (21.2)	108 (49.1)		60 (42.9)	61 (42.7)	
>High school	189 (52.4)	95 (26.2)		139 (62.9)	79 (35.9)		50 (35.7)	16 (11.2)	
Marital status			0.73			0.46			0.70
Married	305 (84.0)	299 (82.1)		196 (88.3)	187 (3.6)		109 (77.3)	112 (78.3)	
Single	18 (5.0)	18 (5.0)		14 (6.3)	16 (7.2)		7 (2.8)	2 (1.4)	
Others	40 (11.0)	47 (12.9)		12 (5.4)	18 (8.1)		28 (19.9)	29 (20.3)	
Occupation			0.04			0.15			0.29
Housewife	198 (54.7)	218 (60.1)		104 (46.9)	112 (50.9)		94 (67.1)	106 (74.1)	
Profession or office worker	85 (23.5)	59 (16.3)		66 (29.7)	48 (21.8)		19 (13.6)	11 (7.7)	
Sales or service	56 (15.5)	51 (14.1)		28 (17.1)	37 (16.8)		18 (12.9)	14 (9.8)	
Others	23 (6.4)	35 (6.9)		14 (6.3)	23 (10.5)		9 (6.4)	12 (8.4)	
Smoking status			0.35			0.25			0.25
Past/never	347 (97.2)	353 (97.0)		211 (95.9)	115 (97.3)		136 (99.2)	138 (96.5)	
Current	10 (2.8)	11 (3.0)		9 (4.1)	6 (2.7)		1 (0.7)	5 (3.5)	
Alcohol intake			0.97			0.43			0.53
Never	191 (52.9)	192 (52.8)		109 (49.3)	103 (46.61)		82 (58.6)	89 (62.2)	
Ever	170 (47.1)	172 (47.3)		112 (50.7)	118 (53.4)		58 (41.4)	54 (37.8)	
Physical activity (MET-min/week)	2425.5 ± 4903.1	2121.3 ± 2772.3	0.31	2669.6 ± 5855.6	1812.2 ± 2330.1	0.046	2021.0 ± 2633.5	2594.7 ± 3290.9	0.11
<396	98 (28.2)	76 (21.0)	0.06	67 (30.9)	55 (25.1)	0.06	31 (23.7)	21 (14.7)	0.27
396–1272	85 (24.4)	100 (27.6)		55 (25.4)	60 (27.4)		30 (22.9)	40 (28.0)	
1272–2772	77 (22.1)	102 (28.2)		39 (18.0)	60 (27.4)		38 (29.0)	42 (29.4)	
≥2772	88 (25.3)	84 (23.2)		56 (25.8)	44 (20.1)		32 (24.4)	40 (28.0)	
Age at menarche (years)	14.5 ± 1.6	14.7 ± 1.9	0.05	14.3 ± 1.5	14.3 ± 1.6	0.77	14.7 ± 1.6	15.4 ± 2.0	0.001
Parity			<0.0001			<0.0001			0.003
Nulliparous	6 (1.8)	39 (10.7)		6 (2.9)	32 (14.5)		0 (0.0)	7 (4.9)	
Primiparous	40 (11.8)	71 (19.5)		26 (12.8)	44 (19.9)		14 (10.4)	27 (18.9)	
Multiparous	293 (86.4)	254 (69.8)		172 (84.3)	145 (65.6)		121 (89.6)	109 (76.2)	
Menopause status			0.88						
Premenopausal	223 (61.3)	221 (60.7)		–	–		–	–	
Postmenopausal	141 (38.7)	143 (39.3)		–	–		–	–	
Age at menopause (years) ^§^	–	–		–	–		48.8 ± 4.2	48.1 ± 5.2	0.23
Type of menopause ^§^									0.02
Natural	–	–		–	–		110 (78.6)	27 (65.5)	
Surgery or others	–	–		–	–		30 (21.4)	36 (34.5)	
Postmenopausal hormone therapy ^§^									0.0004
Yes	–	–		–	–		53 (39.3)	27 (19.6)	
No	–	–		–	–		82 (60.7)	111 (80.4)	
Energy intake (kcal/day)	1632.8 ± 534.8	1722.9 ± 442.9	0.01	1722.5 ± 557.3	1713.3 ± 405.6	0.84	1490.9 ± 464.8	1737.7 ± 496.3	<0.0001
DII score	0.05 ± 2.59	1.03 ± 2.41	<0.0001	0.26 ± 2.57	0.95 ± 2.42	0.004	-0.29 ± 2.60	1.15 ± 2.39	<0.0001

BMI, body mass index; MET, metabolic equivalent unit, MET values are multiples of the resting metabolic rate and were calculated using the short form (version 2.0. April 2004) of the International Physical Activity Questionnaire; DII, Dietary Inflammatory Index. Values are expressed as the mean ± SD or *n* (%); categorical variables were analyzed using the χ^2^ test; continuous variables were analyzed using the Student’s *t*-test. ^§^ In postmenopausal women.

**Table 2:** Dietary Inflammatory Index (DII) component intake of the subjects.

	Total (*n*= 728)			Premenopausal Women (*n* = 444)			Postmenopausal Women (*n* = 284)		
	Controls (*n* = 364)	Cases (*n* = 364)	*p-*Value	Controls (*n* = 223)	Cases (*n* = 221)	*p-*Value	Controls (*n* = 141)	Cases (*n* = 143)	*p-*Value
Carbohydrate (g/d)	187.97 ± 20.01	195.74 ± 19.85	<0.0001	188.56 ± 18.74	192.45 ± 19.92	0.04	187.04 ± 21.92	200.84 ± 18.67	<0.0001
Protein (g/d)	37.30 ± 6.51	34.21 ± 5.56	<0.0001	36.73 ± 6.27	34.69 ± 5.92	0.001	38.20 ± 6.79	33.48 ± 4.86	<0.0001
Total fat (g/d)	17.44 ± 6.02	15.68 ± 6.46	0.0002	17.31 ± 5.58	16.74 ± 6.37	0.32	17.64 ± 6.68	14.03 ± 6.29	<0.0001
MUFAs (g/d)	5.03 ± 2.40	4.54 ± 2.85	0.01	4.86 ± 2.17	4.53 ± 2.52	0.15	5.31 ± 2.71	4.56 ± 3.31	0.04
PUFAs (g/d)	2.87 ± 0.97	2.59 ± 1.07	0.0002	2.80 ± 0.86	2.61 ± 0.98	0.03	2.98 ± 1.12	2.55 ± 1.19	0.002
Saturated fat (g/d)	4.97 ± 2.35	4.46 ± 2.55	0.01	4.78 ± 2.10	4.45 ± 2.36	0.12	5.25 ± 2.69	4.47 ± 2.82	0.02
ω-3 fatty acid (g/d)	0.37 ± 0.24	0.31 ± 0.21	0.0002	0.36 ± 0.22	0.32 ± 0.22	0.10	0.39 ± 0.27	0.28 ± 0.18	0.0001
ω-6 fatty acid (g/d)	2.27 ± 0.71	1.95 ± 0.66	<0.0001	2.25 ± 0.63	2.04 ± 0.67	0.001	2.32 ± 0.82	1.80 ± 0.60	<0.0001
Cholesterol (mg/d)	112.43 ± 57.99	92.73 ± 52.51	<0.0001	109.82 ± 51.84	97.93 ± 52.28	0.02	116.57 ± 66.55	84.69 ± 52.03	<0.0001
Vitamin B1 (mg/d)	0.58 ± 0.13	0.50 ± 0.12	<0.0001	0.58 ± 0.13	0.51 ± 0.12	<0.0001	0.58 ± 0.14	0.48 ± 0.12	<0.0001
Vitamin B2 (mg/d)	0.59 ± 0.18	0.51 ± 0.16	<0.0001	0.57 ± 0.17	0.51 ± 0.16	0.0004	0.62 ± 0.21	0.49 ± 0.12	<0.0001
Vitamin B6 (mg/d)	0.88 ± 0.18	0.80 ± 0.17	<0.0001	0.85 ± 0.17	0.79 ± 0.17	0.0003	0.92 ± 0.19	0.81 ± 0.16	<0.0001
Vitamin B12 (μg/d)	5.11 ± 2.78	3.85 ± 1.80	<0.0001	4.80 ± 2.60	3.91 ± 1.87	<0.0001	5.59 ± 2.99	3.75 ± 1.68	<0.0001
Niacin (mg/d)	8.12 ± 2.03	7.23 ± 1.84	<0.0001	8.01 ± 1.93	7.47 ± 1.99	0.004	8.30 ± 2.18	6.86 ± 1.52	<0.0001
β-carotene (μg/d)	2048.98 ± 1106.03	1688.73 ± 804.25	<0.0001	1951.46 ± 1009.76	1676.17 ± 794.76	0.002	2203.20 ± 1231.23	1708.16 ± 821.13	<0.0001
Vitamin A (μg R.E./d)	384.23 ± 187.41	317.06 ± 137.64	<0.0001	365.96 ± 172.19	317.26 ± 135.91	0.001	413.12 ± 206.59	316.75 ± 140.76	<0.0001
Vitamin C (mg/d)	73.99 ± 33.43	60.63 ± 27.23	<0.0001	74.18 ± 31.08	60.68 ± 27.94	0.0001	77.96 ± 36.59	60.55 ± 26.19	<0.0001
Vitamin D(μg/d)	1.86 ± 1.26	1.55 ± 1.19	0.001	1.74 ± 1.13	1.61 ± 1.28	0.27	2.04 ± 1.44	1.45 ± 1.05	<0.0001
Vitamin E (mg/d)	5.20 ± 1.44	4.61 ± 1.11	<0.0001	5.06 ± 1.29	4.69 ± 1.12	0.001	5.41 ± 1.64	4.48 ± 1.07	<0.0001
Iron (mg/d)	8.24 ± 2.26	7.14 ± 1.48	<0.0001	7.97 ± 2.8	7.11 ± 1.56	<0.0001	8.66 ± 2.33	7.19 ± 1.37	<0.0001
Magnesium (mg/d)	67.96 ± 18.67	62.28 ± 13.48	<0.0001	66.61 ± 17.35	62.66 ± 14.59	0.01	70.10 ± 20.48	61.70 ± 11.58	<0.0001
Zinc (mg/d)	5.75 ± 0.84	5.44 ± 0.82	<0.0001	5.67 ± 0.81	5.42 ± 0.82	0.001	5.89 ± 0.87	5.47 ± 0.84	<0.0001
Selenium (μg/d)	53.93 ± 8.00	51.15 ± 7.63	<0.0001	53.83 ± 7.49	51.78 ± 7.66	0.004	54.07 ± 8.78	50.17 ± 7.51	<0.0001
Folic acid (μg/d)	312.52 ± 100.67	271.50 ± 78.36	<0.0001	301.87 ± 95.52	268.88 ± 79.61	<0.0001	329.36 ± 106.51	275.54 ± 76.48	<0.0001
Fiber (g/d)	12.33 ± 3.93	10.75 ± 2.92	<0.0001	11.81 ± 3.53	10.62 ± 2.95	0.0001	13.16 ± 4.38	10.94 ± 2.87	<0.0001
Flavan-3-ol (mg/d)	6.02 ± 5.07	5.07 ± 5.17	0.01	5.63 ± 5.05	5.17 ± 5.62	0.36	6.65 ± 5.05	4.91 ± 4.41	0.002
Flavones (mg/d)	0.99 ± 0.59	1.03 ± 0.63	0.35	0.94 ± 0.51	1.06 ± 0.68	0.04	1.07 ± 0.70	0.99 ± 0.55	0.30
Flavonols (mg/d)	12.62 ± 8.25	8.99 ± 5.02	<0.0001	11.72 ± 7.19	8.99 ± 5.23	<0.0001	14.05 ± 9.55	9.00 ± 4.69	<0.0001
Flavonones (mg/d)	6.63 ± 7.01	6.64 ± 6.68	0.98	6.60 ± 6.41	6.91 ± 6.92	0.62	6.68 ± 7.90	6.23 ± 6.30	0.60
Anthocyanidins (mg/d)	2.48 ± 1.70	1.80 ± 1.37	<0.0001	2.15 ± 1.47	1.67 ± 1.25	0.0003	3.00 ± 1.91	1.99 ± 1.53	<0.0001
Isoflavones (mg/d)	9.44 ± 7.27	8.14 ± 5.31	0.01	8.62 ± 5.81	8.11 ± 5.23	0.33	10.72 ± 8.98	8.19 ± 5.46	0.005
Garlic (g/d)	0.87 ± 0.55	0.56 ± 0.32	<0.0001	0.85 ± 0.55	0.58 ± 0.35	<0.0001	0.90 ± 0.56	0.54 ± 0.26	<0.0001
Ginger (g/d)	0.003 ± 0.01	0.003 ± 0.01	0.91	0.004 ± 0.01	0.004 ± 0.01	0.86	0.003 ± 0.01	0.002 ± 0.01	0.65
Onion (g/d)	8.17 ± 4.83	6.88 ± 3.67	<0.0001	8.19 ± 4.71	6.95 ± 3.81	0.002	8.12 ± 5.02	6.76 ± 3.44	0.01
Green tea (g/d)	29.14 ± 48.17	45.99 ± 87.65	0.001	29.22 ± 47.85	52.34 ± 96.11	0.002	29.01 ± 48.83	36.17 ± 71.91	0.33
Pepper (g/d)	0.025 ± 0.02	0.019 ± 0.02	0.0002	0.025 ± 0.02	0.019 ± 0.02	0.01	0.024 ± 0.02	0.018 ± 0.02	0.01
Alcohol (g/d)	7.78 ± 94.52	2.30 ± 11.97	0.27	10.95 ± 120.29	2.88 ± 14.74	0.32	2.78 ± 13.35	1.41 ± 5.33	0.26

MUFAs, monounsaturated fatty acids; PUFAs, polyunsaturated fatty acids. Values are expressed as the mean ± SD represented per 1000 kcal; variables were analyzed using the Student’s *t*-test.

**Table 3:** Odds ratios (95% CIs) of breast cancer and Dietary Inflammatory Index (DII) score.

	Tertiles of DII			*p-*Trend ^‡^
	T1	T2	T3	
Total (*n* = 728)				
Median (range)	−2.74 (≤−1.52)	−0.01 (−1.51–1.23)	2.91 (>1.23)	
No. of controls/cases	121/56	122/141	121/167	
Crude OR (95% CI)	Ref.	2.50 (1.68–3.72)	2.98 (2.01–4.42)	<0.0001
Adjusted OR (95% CI) ^a^	Ref.	2.94 (1.87–4.63)	3.68 (2.34–5.80)	<0.0001
Premenopausal women (*n* = 444)				
Median (range)	−2.37 (≤−1.10)	0.13 (−1.09–1.45)	3.20 (>1.45)	
No. of controls/cases	74/46	75/85	74/90	
Crude OR (95% CI)	Ref.	1.82 (1.13–2.95)	1.96 (1.21–3.16)	0.01
Adjusted OR (95% CI) ^b^	Ref.	1.87 (1.08–3.24)	2.11 (1.21–3.67)	0.01
Postmenopausal women (*n* = 284)				
Median (range)	−2.94 (≤−1.96)	−0.06 (−1.95–1.02)	2.62 (>1.02)	
No. of controls/cases	47/19	47/49	47/75	
Crude OR (95% CI)	Ref.	2.58 (1.32–5.02)	3.95 (2.07–7.53)	<0.0001
Adjusted OR (95% CI) ^c^	Ref.	3.21 (1.35–7.23)	6.13 (2.66–14.12)	<0.0001

DII, Dietary Inflammatory Index; Ref., reference category; ORs, odds ratios; CIs, confidence intervals. ^a^ Total was adjusted for height, education level, occupation, age at menarche, parity, total energy intake, and BMI; ^b^ Premenopausal women were adjusted for education level, physical activity, parity, and BMI; ^c^ Postmenopausal women were adjusted for education level, age at menarche, parity, total energy intake, BMI, dietary supplement use, menopause type, and postmenopausal hormone use. **^‡^** Linear trends across categories of DII score were tested using the median value for each category.

**Table 4:** Odds ratios (95% CIs) of breast cancer characterized by hormone receptor status according to DII scores.

	No. of Controls	ER+/PR+				ER−/PR−			
		No. of Cases	Median	Crude OR (95% CI)	Adjusted OR (95% CI)	No. of Cases	Median	Crude OR (95% CI)	Adjusted OR (95% CI)
Total ^a^									
T1 (≤−1.52)	121	28	−2.75	Ref.	Ref.	14	−2.75	Ref.	Ref.
T2 (−1.51–1.23)	122	91	0.05	3.22 (1.97–5.28)	3.88 (2.21–6.79)	31	0.03	2.20 (1.11–4.33)	2.56 (1.18–5.55)
T3 (>1.23)	121	100	2.80	3.57 (2.19–5.82)	4.29 (2.45–7.54)	40	2.88	2.86 (1.48–5.52)	5.00 (2.32–10.77)
*p*-Trend ^‡^				<0.0001	<0.0001			<0.0001	<0.0001
Premenopausal women ^b^									
T1 (≤−1.10)	74	26	−2.52	Ref.	Ref.	12	−2.37	Ref.	Ref.
T2 (−1.09–1.45)	75	67	0.16	2.54 (1.46–4.43)	2.48 (1.32–4.65)	11	0.11	0.90 (0.38–2.18)	0.94 (0.35–2.51)
T3 (>1.45)	74	68	3.03	2.62 (1.50–4.56)	2.59 (1.37–4.88)	14	3.18	1.17 (0.51–2.69)	1.64 (0.62–4.33)
*p*-Trend ^‡^				0.002	0.01			0.69	0.28
Postmenopausal women ^c^									
T1 (≤−1.96)	47	6	−2.97	Ref.	Ref.	6	−3.00	Ref.	Ref.
T2 (−1.95–1.02)	47	22	−0.03	3.67 (1.36–9.85)	5.96 (1.63–21.84)	15	−0.03	2.50 (0.89–7.00)	2.62 (0.73–9.47)
T3 (>1.02)	47	30	2.41	5.00 (1.90–13.12)	11.00 (2.93–41.30)	27	2.55	4.50 (1.70–11.90)	5.22 (1.55–17.55)
*p*-Trend ^‡^				<0.0001	0.0004			0.002	0.01

DII, Dietary Inflammatory Index; Ref., reference category; ORs, odds ratios; CIs, confidence intervals; ER+, estrogen receptor positive; ER−, estrogen receptor negative; PR+, progesterone receptor positive; PR−, progesterone receptor negative. ^a^ The total study sample was adjusted for height, education level, occupation, age at menarche, parity, total energy intake and BMI; ^b^ Premenopausal women were adjusted for education level, physical activity, parity, and BMI; ^c^ Postmenopausal women were adjusted for education level, age at menarche, parity, total energy intake, BMI, dietary supplement use, menopause type, and postmenopausal hormone use. ^‡^ Linear trends across categories of DII score were tested using the median value for each category.
